# CGRP and migraine from a cardiovascular point of view: what do we expect from blocking CGRP?

**DOI:** 10.1186/s10194-019-0979-y

**Published:** 2019-03-12

**Authors:** Valentina Favoni, Luca Giani, Linda Al-Hassany, Gian Maria Asioli, Calogera Butera, Irene de Boer, Martina Guglielmetti, Chrysoula Koniari, Theodoros Mavridis, Marge Vaikjärv, Iris Verhagen, Angela Verzina, Bart Zick, Paolo Martelletti, Simona Sacco, European Headache Federation School of Advanced Studies (EHF-SAS)

**Affiliations:** 1 Department of Biomedical and Neuromotor Sciences, University of Bologna, Bologna, Italy; 2 IRCCS Istituto delle Scienze Neurologiche di Bologna, Via Altura, 3 Pad. G, 40139 Bologna, Italy; 3 Ricovero Ferdinando Uboldi, Paderno Dugnano, Italy; 4 Division of Vascular Medicine and Pharmacology, Department of Internal Medicine, Erasmus MC, Rotterdam, The Netherlands; 5 Dipartimento Neurologico e INSPE, IRCCS Ospedale San Raffaele, Milan, Italy; 6 Department of Neurology, Leiden University Medical Center, Leiden, The Netherlands; 7 Department of Clinical and Molecular Medicine, Sapienza University, Rome, Italy; 8 Regional Referral Headache Center, Sant’Andrea Hospital, Rome, Italy; 9 Department of Clinical Pathology, University of Sassari, Sassari, Italy; 10 1st Neurology Department, Aeginition Hospital, School of Medicine, National and Kapodistrian University of Athens, Athens, Greece; 11 Faculty of Medicine, University of Tartu, Tartu, Estonia; 12 Neurology Clinic, University of Perugia, Perugia, Italy; 13 S. Maria della Misericordia Hospital, Perugia, Italy; 14 UOC Neurologia e Stroke Unit, Ospedale SS Filippo e Nicola, Avezzano, Italy; 15 Department of Applied Clinical Sciences and Biotechnology, University of L’Aquila, L’Aquila, Italy

**Keywords:** CGRP, CGRP antibody, Migraine treatment, Cardiovascular

## Abstract

Calcitonin gene-related peptide (CGRP) is a neuropeptide with a pivotal role in the pathophysiology of migraine. Blockade of CGRP is a new therapeutic target for patients with migraine. CGRP and its receptors are distributed not only in the central and peripheral nervous system but also in the cardiovascular system, both in blood vessels and in the heart. We reviewed the current evidence on the role of CGRP in the cardiovascular system in order to understand the possible short- and long-term effect of CGRP blockade with monoclonal antibodies in migraineurs.

In physiological conditions, CGRP has important vasodilating effects and is thought to protect organs from ischemia. Despite the aforementioned cardiovascular implication, preventive treatment with CGRP antibodies has shown no relevant cardiovascular side effects. Results from long-term trials and from real life are now needed.

## Introduction

Migraine is one of the leading chronic neurological disorders, considered among the top five causes of long-term disability and affecting 15% of the population, mainly women [1, 2]. Treatments for migraine can be divided into abortive and prophylactic therapy. Calcitonin gene-related peptide (CGRP) blockade has emerged as a therapeutic target for migraine. CGRP is a neuropeptide released from perivascular nerve fibers after trigeminal nerve activation performing a pivotal role in the pathophysiology of migraine [3, 4]. In recent years, monoclonal antibodies against CGRP and its receptors have been developed and tested in clinical trials involving migraine patients. The site of action of these antibodies is still debated. Because they are large molecules, they have limited potential to pass the blood-brain barrier (BBB) and may act at the peripheral level. However, some studies have shown that brain structures involved in the pathophysiology of migraine (e.g. trigeminal ganglion and the paraventricular structures within the brain stem) are not fully protected by the BBB [5–7], hence effective migraine treatment drugs need not to pass through the BBB. Furthermore, the antimigraine action site may reside in areas not protected by the BBB such as the intra- and extracranial vessels, dural mast cells, and the trigeminal system [3]. Interestingly, CGRP receptors are located not only in the central and peripheral nervous system but also in the cardiovascular system including blood vessels and the heart [8]. CGRP acts as a very potent vasodilator and plays an important role in regulating vascular resistance and regional organ blood flow in physiological and also during pathological conditions like cerebral or cardiac ischemia [7, 9–11]. We reviewed the current evidence on the role of CGRP in the cardiovascular system to understand the possible short- and long-term effect of CGRP blockade with monoclonal antibodies in migraineurs.

## Methods of review

Two independent reviewers conducted an independent search on PubMed on July 20th, 2018 using the search terms “cgrp” AND “cardiovascular system” OR “cardiovascular” AND “system”. This search generated 1585 abstracts, which were reviewed independently, and articles were selected on the basis of relevance to the present topic. Discrepancies between investigators were rechecked and, if necessary, discussed with a third investigator until consensus was achieved. Every author added additional papers when needed in their respective section. The final reference list was generated on the basis of originality and relevance to the topic of this Review.

### Calcitonin gene-related peptide and CGRP receptors

CGRP, a peptide with 37 amino acid residues, exists in humans in two isoforms, α and βCGRP, otherwise known as CGRP I and II. Alternative splicing of the *CACL1* gene (calcitonin gene) produces, most prominently in the central and peripheral nervous system, αCGRP [12, 13]. Transcription of the *CACLII* gene leads to βCGRP, most abundantly found in the enteric sensory system [12, 13]. αCGRP and βCGRP share > 90% homology in humans (with only three amino acids being different) [14]. Therefore, it is logical that their biological activity is similar. CGRP is expressed in the peripheral nervous system in thin unmyelinated C fibers, and at numerous sites in the central nervous system [4, 15–17].The synthesis and release of CGRP can be triggered by activation of the transient receptor potential vanilloid subfamily member 1 (TRPV1). One of the ligands of TRPV1, capsaicin, was first used to demonstrate the release of CGRP from sensory neurons [10]. However, the synthesis and release of CGRP is mediated by many factors, which are still being investigated.

CGRP acts by activating multiple receptors [18–20]. The functional CGRP receptor consists of three components: calcitonin-like receptor (CLR), receptor component protein (RCP) which defines the G-protein to which the receptor binds, and receptor activity-modifying protein 1 (RAMP1) [19–21]. RCP links the receptor to an intracellular C protein-mediated signaling pathway, which increases cyclic adenosine monophosphate (cAMP) levels [22]. For updated classification and nomenclature of calcitonin/CGRP family of peptides and receptors see Table 1. CGRP receptors are also present on the smooth muscle cells of human cranial and coronary arteries [9, 23]. It remains unclear if there is a difference in the expression of CGRP receptors between cranial and coronary arteries, but functional studies suggest a higher expression of CGRP receptors in cranial arteries. Receptor components of CGRP have also been identified in the trigeminal ganglion, cerebral cortex, hippocampus, thalamus, hypothalamus, brainstem, spinal cord and cerebellum [24–26]. As such, CGRP probably has both neural and vascular actions.

**Table 1:**
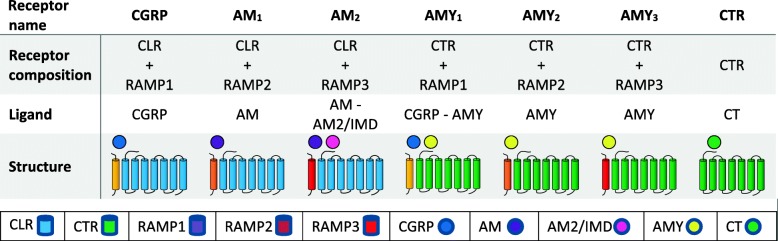
Current classification of human calcitonin-family receptors, subunit composition and respective ligands

*CGRP* Calcitonin Gene-Related Peptide, *AM* Adrenomedullin, *AMY* Amylin, *CTR* Calcitonin Receptor, *CLR* Calcitonin receptor-like receptor, *RAMP* receptor activity-modifying proteins, *AM2/IMD* Adrenomedullin 2/Intermedin

### Endothelial dysfunction and CGRP in migraineurs

Various vascular mechanisms have been described in order to explain the role of CGRP in vasodilation of peripheral vascular beds. The presence of an NO- and endothelium-independent pathway, which leads to vascular relaxation, has been observed in smooth muscle cells of most tissues [27, 28]. However, CGRP also has the capability to stimulate the production of NO by acting via a receptor located on the endothelium. This endothelium-dependent relaxation pathway results in an accumulation of cAMP and production of NO through endothelial protein kinase A/endothelial NO Synthase (PKA/eNOS) signaling. Eventually, NO diffuses into adjacent smooth muscle cells and activates guanylate cyclase. This finally leads to the production of cGMP and relaxation of vessels [11, 28, 29]. The role of endothelium in migraine pathophysiology is still debated. Some studies indicate that migraineurs have an impaired arterial and endothelial function as compared to non-migraineurs [30]. On the contrary, a recent study suggested that the contribution of endothelium to CGRP-induced vasodilation may not be significant [31]. In fact, cutaneous microvascular sensitivity to endothelial and non-endothelial donors including CGRP showed no difference between a group of patients with migraine compared to controls [32]. It has been speculated that alterations at the endothelial level may contribute to the increased risk (approximately 50%) of several cardiovascular diseases such as ischemic and hemorrhagic stroke, angina and myocardial infarction, which has been observed in several studies that compared migraineurs (with aura) to non-migraineurs [33–38].

### Physiological and pathological influence of CGRP on the cardiovascular system

CGRP release induces relaxation of smooth muscle cells due to an increase in cAMP and leads to activation of protein kinase A, which phosphorylates and opens potassium channels [39, 40]. In blood vessels, CGRP acts as an extremely potent vasodilator when compared to several known vasodilators such as histamine, prostaglandin E2 and substance P [41]. Even so, CGRP seems to have no pivotal role in the physiological regulation of systemic blood pressure. For instance, blocking CGRP does not affect systemic blood pressure in healthy volunteers [42]. In the heart, CGRP is localized in sensory nerve fibers and around peripheral arteries [9]. There are specific binding sites for CGRP linked to stimulation of adenylate cyclase activity more concentrated in the atrium [43]. In both rats and humans, in addition to its vasodilator effect, intravenous CGRP administration has been shown to cause positive inotropic and chronotropic effects on the heart [44–47]. In physiological conditions, CGRP might act on a more local level, regulating vascular responsiveness and protecting organs from injury. Thus, CGRP may have a cardiovascular protective role. In pathophysiological situations, like hypertension, conflicting observations have been made. Both decreased, increased and unchanged plasma levels of CGRP have been observed in patients with essential hypertension [48, 49]. While CGRP does not seem to be involved in the physiological regulation of blood pressure, it has a protective role against the development of hypertension. It exerts its action mainly directly on smooth muscle cells in the vessel wall, most prominently in the microvasculature, which is responsible for the majority of the peripheral vascular resistance and thus, the blood pressure [9, 50].

Moreover, CGRP given intravenously to patients with congestive heart failure improved myocardial contractility without any consistent change in arterial pressure or heart rate [51]. CGRP causes beneficial effects on physiological cardiac hypertrophy helping the heart to distinguish physiological, exercise-induced from pathological stresses [52].

In addition, CGRP may play an important role in mediation of ischemic preconditioning, the phenomenon in which a tissue is rendered resistant to the deleterious effects of prolonged ischemia. Capsaicin, which evokes CGRP release from sensory nerves, is reported to protect against myocardial injury by ischemia-reperfusion in the isolated perfused rat heart [53]. Moreover, pretreatment with CGRP for 5 min produces a significant protective effect on the ischemic myocardium, as shown by the enhanced post-ischemic myocardial function, the reduced incidence of ventricular arrhythmia, and the attenuated release of creatine phosphate kinase [54]. Some studies have also suggested that the protective role of CGRP against ischemia may be due to induced vasodilation [55]. In the setting of brain ischemia, it might reduce the extent of the infarct zone [56], while in the case of subarachnoid hemorrhage, there is evidence that CGRP is protective against cerebral vasospasm [57–59]. CGRP might be protective also in the setting of chronic cerebrovascular disease (as induced by bilateral carotid stenosis) and the subsequent neuronal injury and cognitive impairment [56].

### Sex differences and CGRP pathophysiology

CGRP plasma levels are higher in women than in men [60]. Cardiovascular benefits of CGRP, such as vasodilatory and hypotensive effects on the arteries [61] and the positive inotropic effects on the myocardium are strongly influenced by fluctuations in female sex hormone levels [62]. Furthermore, sex hormone receptors are found in the trigeminovascular and cardiovascular system and, therefore, it is likely that there is an interaction between female sex hormones and CGRP, but the exact mechanism is still not fully understood [63, 64]. In animal models, females had higher CGRP levels in the medulla and lower expression of CLR, RAMP1 and RCP-encoding mRNA in tissues, compared to males, suggesting that CGRP receptor synthesis, expression or release in the trigeminovascular system may be regulated by fluctuating female sex hormones. Numerous animal and human studies have shown that cyclic fluctuations of ovarian hormones (mainly estrogen) modulate CGRP both in peripheral and central nervous system [65–67]. It is, therefore, reasonable to think that females, in particular, are sensitive to therapeutic effects of CGRP blockade, but also to adverse events. In clinical practice, it would be useful to know whether female migraineurs have an additional higher cardiovascular risk if they are prescribed CGRP monoclonal antibodies for the treatment of migraine. Future studies should assess possible sex differences in the benefits and harms of drugs acting on the CGRP and its receptor.

### Blocking CGRP

The blockade of the CGRP system has been obtained by different molecules: non-peptide CGRP antagonists also known as “gepants” (olcegepant, telcagepant, ubrogepant, atogepant), monoclonal antibodies against CGRP (eptinezumab, fremanezumab, galcanezumab) and monoclonal antibodies against CGRP receptor (erenumab).

Gepants have demonstrated efficacy in relieving migraine in clinical trials and do not cause direct vasoconstriction. However, olcegepant had to be administered intravenously due to its low oral bioavailability [68, 69]. Encouraged by the efficacy of blocking CGRP for the treatment of migraine, monoclonal antibodies able to block either CGRP or its receptor were developed. CGRP antibodies have a slower onset of action compared with the CGRP receptor antagonists, which is consistent with the idea of a slower penetration into the interstitial space of the vascular smooth muscle tissue. The inhibition is evident one week after dosing [70]. Moreover, CGRP antibodies might scavenge CGRP for up to 1.5 months [7].

### Short-term effects of blocking CGRP

The cardiovascular safety of short-term CGRP blockade has been widely explored for both CGRP antagonists and for monoclonal antibodies. In animal models, several studies conducted on non-peptidic CGRP-R antagonists (olcegepant) evidenced that short-term blockade of CGRP have no effects on hemodynamic parameters such as heart rate, blood pressure, cardiac output, coronary flow or severity of ischemia were observed in different animal species [71–73]. CGRP antagonism is safe in healthy volunteers; a study demonstrated that the administration of telcagepant at supra-therapeutic dosage did not induce vasoconstriction both in peripheral and central vascular beds in healthy men [74]. Moreover, this drug did not influence treadmill-exercise-time in patients with stable angina [75].

Clinical trials of single-doses of oral telcagepant administered for acute treatment of migraine showed a total absence of cardiovascular side effects in migraine patients [76, 77]. Only minor adverse events were registered (dry mouth, somnolence, dizziness, nausea, fatigue) [78].

Since the half-life of monoclonal antibodies is longer (21–50 days) [79] than that of non-peptidic CGRP antagonists, the blockade of CGRP has a longer duration. In rats CGRP blocking antibodies inhibit the neurogenic vasodilation, confirming the role of these molecules in treating migraine, but no effect on heart rate and arterial blood pressure was observed [70]. Similar results were obtained using fremanezumab in monkeys, where the effect of single or multiple (once weekly for 14 weeks) injections on cardiovascular parameters were evaluated. No meaningful modifications of ECG parameters, heart rate, and systolic blood pressure were observed in both situations [80]. In another trial, healthy women over 40 years old (mean age 56 years) were monitored for 24 weeks after administration of a single dose of fremanezumab at different dosages. No changes in ECG parameters, nor heart rate or blood pressure were registered [81].

Safety and tolerability data from clinical trials are encouraging for the anti-CGRP monoclonal antibodies for the treatment of both episodic and chronic migraine. All phase II and phase III clinical trials completed so far for the four developed monoclonal antibodies did not show any safety problem concerning the cardiovascular system [82, 83]. It must be noted that the patients recruited for clinical trials were young (age range 18–65, with a mean of about 40 years) usually without any significant cardiovascular disease. Therefore, the safety profile of this class of drugs in high-risk patients has to be specifically addressed. A randomized, double-blind placebo-controlled study was performed for studying the cardiovascular effect of erenumab in patients with stable angina. In particular, the investigators evaluated the impact of a dose of the drug (iv infusion of 140 mg) on exercise time during a treadmill test. There was no decrease in treadmill test, so they concluded that the inhibition of CGRP receptor does not worsen myocardial ischemia [84]. One major criticism about this study regards the population selected, which was composed of non-migraineurs; data indicate that migraineurs are at risk for cardiovascular events [34, 36]. Thus, safety of anti-CGRP monoclonal antibodies in migraineurs may be different from that of the general population. Additionally, in that study most patients (80%) were males, while migraine is more prevalent in women. As previously discussed, sex hormones influence the activity of CGRP on the vascular tone and female migraineurs are at increased risk of myocardial infarction [85], possibly exposing them to a specific sensitivity to CGRP blockade [77].

### Long-term effects of blocking CGRP

Pre-registration trials are mostly limited to a maximum of 6 months. Considering the role of CGRP in cardiovascular physiology and in the pathophysiology, this time frame could not be enough to exclude effects of blockade in the long run. There is just one published article about a trial longer than 6 months using anti-CGRP drugs [86]. The interim analysis after one year of open label extension of an erenumab trial (EudraCT 2012–005331-90, NCT01952574) among 383 subjects exposed for a median of 575 days reported one case of death in a 52-year-old man with pre-existing cardiovascular risk factors (hypertension, hypercholesterolemia, obesity, familial history) and post-mortem evidence of severe coronary atherosclerosis and use of sympathomimetics. A case of transient exercise-induced myocardial ischemia during a treadmill test was confounded by sumatriptan intake 4 h prior to the event [86]. Considering the presence of confounding factors, these adverse events may be not related to the treatment. However, a limitation of the study is the lack of a placebo group, which makes it difficult to differentiate spontaneously occurring adverse events from adverse events due to erenumab.

In all short- and long-term studies published, investigators have not observed any hypertensive effect of anti-CGRP drugs, nor were any negative effects observed regarding the development or aggravation of cardiac failure, although this last issue was not specifically addressed, there was no specific monitoring, and it is not clear if any patient with heart failure was treated. Moreover, the time frame might be not enough to observe a clinical effect of organ remodeling.

Regarding the cerebrovascular risk of anti-CGRP drugs, no safety issues have emerged from all the trials completed so far.

## Conclusions

In conclusion, CGRP plays an important role in migraine but also in physiological and pathological cardiovascular conditions. We can speculate that CGRP may act as a link between the brain and the heart. Data emerging from trials with CGRP antibodies suggest that this specific blockade of the CGRP pathway is a safe treatment. To our knowledge, no serious adverse events have been reported since approval of anti-CGRP monoclonal antibodies for migraine treatment in May 2018. However, results from long-term trials and real life are particularly awaited in order confirm these encouraging data on the long-term safety of the new migraine preventive drugs.
